# Chronic musculoskeletal pain prospectively predicts insomnia in older people, not moderated by age, gender or co-morbid illnesses

**DOI:** 10.1038/s41598-021-81390-6

**Published:** 2021-01-15

**Authors:** Regina Wing Shan Sit, Benjamin Hon Kei Yip, Bo Wang, Dicken Cheong Chun Chan, Dexing Zhang, Samuel Yeung Shan Wong

**Affiliations:** Jockey Club School of Public Health and Primary Care, The Chinese University of Hong Kong, 4/F, School of Public Health, Prince of Wales Hospital, Shatin, NT Hong Kong

**Keywords:** Health care, Medical research

## Abstract

The study evaluated if chronic musculoskeletal (MSK) pain predicts the severity of insomnia, and whether the effect is moderated by age, gender, and number of comorbid diseases in older people. An 18-month prospective study was performed within the framework of a community health program in Hong Kong. A total of 498 older people aged ≥ 60 with multimorbidity were recruited. The predictors included the presence of chronic MSK pain, pain measured by the Brief Pain Inventory (BPI), insomnia measured by baseline Insomnia Severity Index (ISI), and number of co-morbid diseases, age, and gender. The outcome was ISI repeated at 18 months. The moderators included age, gender, and number of comorbid diseases. Multivariate linear regression and moderation analysis were conducted. We found that the presence of chronic MSK pain (β = 1.725; 95% CI, 0.607–2.842; P < 0.01) predicted the severity of ISI, after controlling for age, gender, BMI, and the number of comorbid diseases. Participants with chronic MSK pain throughout the period had worse trend of improvement in ISI compared to those who were “pain-free” (β = 2.597; 95% CI, 1.311–3.882; P < 0.001). Age, gender, and number of comorbid diseases did not moderate the longitudinal relationship. We propose that pain management should prioritized in the prevention of insomnia.

## Introduction

Chronic musculoskeletal (MSK) pain is very common among older people, with varying impacts on functional, psychological, and social impairment^1^. The World Health Organization (WHO) 2015 reported that MSK conditions posed a threat to the healthy aging population, and leaht to significant socioeconomic burden^2^. Painful MSK conditions have been associated with a reduced capacity to engage in physical activity, and it further causes functional decline, frailty, reduced well-being, loss of independence, and depressive symptoms^3^. Chronic MSK pain is one of the most common reasons for seeking medical help, but is often under-recognized and undertreated^4–6^. More importantly, the impact of chronic pain is profound across the lifespan. The Global Disease Burden (GBD) has reported that MSK disorders contributes to 17% of the global years-lived with disability^7^, and is one of the major causes of work loss and early retirement^8,9^.

Numerous studies have been conducted to evaluate the relationship between chronic pain and sleep; however, the direction of causality remains unclear, as most reported studies are cross-sectional^10^. Among those reported prospective studies, the conclusion is sleep impairment is a stronger and more reliable predictor of pain, than pain is a predictor of sleep impairment; yet, most of the included studies used sleep impairment as the predictor of pain, and only a few studies used pain as the predictor of sleep impairment^10,11^. One study conducted in adolescents with different bodily pain, using a 10-day actigraphy, concluded that pain did not predict sleep quality or efficiency^12^. Another study conducted in a middle-aged population with mixed bodily pain, using a 7-day actigraphy, also concluded that pain was not a reliable predictor of subsequent sleep impairment^13^. To the best of our knowledge, only 2 studies have suggested that pain as a predictor of sleep impairment. In one study, low back pain intensity was identified as a predictor of sleep disturbance using a 7-day Armband electronic device^14^, while the other study showed that peri-menopausal women who reported night pain have less sleep efficiency in a 27-day actigraphy^15^. One possible reason that pain predicts sleep impairment in adults but not among adolescents is the different sleep pattern across the lifespan of healthy individuals, with significantly increased sleep latency, stage 1 and stage 2 sleep, and reduced rapid-eye movement sleep in adults^16^. Nevertheless, unlike the ample evidence that sleep is prospectively associated with pain-related outcomes in different populations^17^, longitudinal prospective data with pain as the predictor of sleep impairment is lacking. This has led to controversies regarding the bidirectional relationship between pain and sleep^11^.

Moreover, a wealth of data has suggested that pain and sleep disturbance vary according to the most prominent sociodemographic factors, namely, age and gender^11,18^. Both pain and sleep impairment tend to increase with age and among female populations^3,19^. A previous cross-sectional moderation analysis concluded that female gender with insomnia is associated with greater pain^20^. Yet, whether age and gender moderate the relationship between pain and sleep impairment in a longitudinal direction remains largely unknown. Furthermore, the moderating effect of multimorbidity, which is common in older people and is associated with pain and sleep disorder, has never been explored in a longitudinal pain-sleep relationship.

Therefore, this study aimed to evaluate whether chronic MSK pain predicts insomnia in a longitudinal direction, and whether the effect is moderated by age, gender, and the number of comorbid illnesses. We also investigated whether the number of reported pain sites, pain severity, and interferences, are associated with the severity of insomnia.

## Results

718 older adults completed the baseline assessment and 498 of them completed the follow-up assessment at 18 months. 220 declined the telephone interviews or could not be contacted.

The background characteristics of 498 participants were summarized in Table 1. It included 364 women (73.1%), with a mean age of 69.19 ± 6.26 years, BMI of 23.96 ± 3.55 kg/m^2^, the presence of 4.18 ± 1.85 comorbid medical conditions, and a baseline ISI score of 11.62 ± 4.85. Among these participants, 391 (78.5%) reported chronic MSK pain, with an average of 3.76 ± 2.38 pain sites, a mean BPI severity score of 4.44 ± 1.88, and mean BPI interference score of 2.92 ± 2.09. Statistically significant baseline differences between the MSK pain and non-pain groups (P < 0.001) were detected for female gender, the number of comorbid diseases, and baseline insomnia scores (Table 1).

**Table 1:** Baseline background Data (N = 498) stratified by the presence of chronic MSK pain^^^ (Mean ± standard deviation (SD) / count (col %)).

	Total (N = 498)	MSK Pain (N = 391)	No MSK pain (N = 107)	P-value
Age, (years)	69.19 ± 6.26	68.98 ± 6.24	69.93 ± 6.30	0.164
Gender				
Female	364 (73.1%)	305 (78%)	59 (55.1%)	< 0.001
Male	134 (26.9%)	86 (22%)	48 (44.9%)	
BMI	23.96 ± 3.55	24.05 ± 3.50	23.60 ± 3.73	0.248
No. of comorbid diseases	4.18 ± 1.85	4.37 ± 1.91	3.47 ± 1.36	< 0.001
2	98 (19.7%)	69 (17.6%)	29 (27.1%)	< 0.001
3	112 (22.5%)	78 (19.9%)	34 (31.8%)	
≥ 4	288 (57.8%)	244 (62.4%)	44 (41.1%)	
ISI	11.62 ± 4.85	12.14 ± 4.84	9.73 ± 4.40	< 0.001
No. of pain sites	–	3.76 ± 2.38	–	–
BPI-severity	–	4.44 ± 1.88	–	–
BPI-interference	–	2.92 ± 2.09	–	–

^^^Abbreviations: BMI: Body Mass Index; BPI: Brief Pain Inventory; ISI: Insomnia Severity Index; MSK: Musculoskeletal.

**Figure 1:**
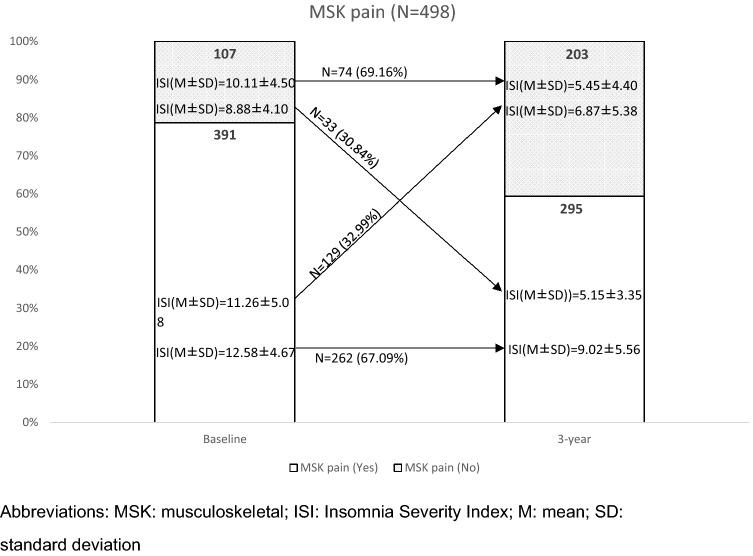
The transition of MSK pain from baseline to 18-month later among 498 people and the corresponding ISI. Abbreviations: MSK: musculoskeletal; ISI: Insomnia Severity Index; M: mean; SD: standard deviation.

The ISI of the MSK pain and non-pain groups at 18 months were 8.59 ± 5.49 and 6.35 ± 5.09, respectively. Throughout the 18 months, 33 participants developed chronic MSK pain (no pain-pain), and 129 participants recovered from their pain (pain-no pain). Majority of the participants (n = 262) stayed in the chronic MSK pain group (pain-pain) and had worse ISI. Only 74 participants remained in the pain-free group (no pain-no pain). The corresponding ISI of each pain transition groups is shown in Fig. 1.

In the multivariate analysis, the presence of chronic MSK pain (β = 1.725; 95% CI, 0.607–2.842; P < 0.01), female gender (β = 1.417; 95% CI 0.397–2.436; P < 0.01), and baseline ISI (β = 0.399; 95% CI 0.305–0.494; P < 0.001), were found to predict the severity of ISI, after controlling for age, gender, BMI and the number of co-morbid diseases (Table 2). The longitudinal relationship was further analyzed; participants in the “pain-pain” group had the worse trend of improvement compared to those in the “no pain-no pain” group (β = 2.597; 95% CI 1.311–3.882; P < 0.001). No statistical significance was detected between other groups. (Table 3).

**Table 2:** Association of the predictive factors and insomnia at 18 months (n = 498).

Variables	Estimated beta coefficient (95% CI)^^^			
	Partial adjusted model^†^	P-value	Fully adjusted model^Ψ^	P-value
Age	− 0.001 (− 0.072, 0.069)	0.977	0.014 (− 0.056, 0.084)	0.691
Female	1.709 (0.716, 2.703)	< 0.01	1.417 (0.397, 2.436)	< 0.01
BMI	− 0.022 (− 0.146, 0.103)	0.733	− 0.030 (− 0.152, 0.093)	0.636
No. of chronic illnesses	− 0.005 (− 0.250, 0.240)	0.966	− 0.032 (− 0.280, 0.215)	0.798
MSK pain (Yes)	1.959 (0.879, 3.039)	< 0.001	1.725 (0.607, 2.842)	< 0.01
Insomnia (baseline)	0.447 (0.357, 0.538)	< 0.001	0.399 (0.305, 0.494)	< 0.001

^^^Abbreviations: CI, confidence interval; MSK: Musculoskeletal; BMI: Body Mass Index;

^†^Adjusting for insomnia at baseline except for the model of insomnia at baseline on insomnia at 3 years;

^Ψ^Adjusting for age, gender, BMI, and no. of comorbid diseases.

**Table 3:** Association of MSK pain transition groups and insomnia at 18 months (n = 498).

MSK pain^Ψ^		N	Estimated beta coefficient (95% CI) ^^^			
			Partial adjusted model 1^†^		Fully adjusted model 2^‡^	
Baseline	18 months		β (95% CI)	P-value	β (95% CI)	P-value
No	No	74	Ref	–	Ref	–
No	Yes	33	− 1.422 (− 3.205, 0.362)	0.118	0.194 (− 1.822, 2.210)	0.850
Yes	No	129	− 0.875 (− 1.877, 0.127)	0.087	0.963 (− 0.443, 2.370)	0.179
Yes	Yes	262	2.029 (1.147, 2.911)	< 0.001	2.597 (1.311, 3.882)	< 0.001

^^^Abbreviations: CI, confidence interval; MSK, musculoskeletal; N, sample size; β, beta coefficient;

^Ψ^No, people without MSK pain; Yes, people with MSK pain;

^†^Adjusting for insomnia at baseline.

^‡^Adjusting for age, gender, BMI, no. of comorbid diseases and insomnia at baseline.

Among those with reported chronic MSK pain at 18 months (N = 248), the BPI- severity and interference scores were positively associated with ISI (β = 0.544; 95% CI 0.178–0.911; P < 0.01 and β = 0.764; 95% CI 0.443–1.084; P < 0.001, respectively) .This association was not found in the number of pain sites (Table 4).

**Table 4:** Association of pain score with insomnia among participants with MSK pain (n = 248) at 18 months.

Variables	Estimated beta coefficient (95% CI)^^^			
	Univariate model	P-value	Fully adjusted model^†^	P-value
No. of pain sites	0.160 (− 0.141, 0.461)	0.296	− 0.120 (− 0.407, 0.167)	0.410
BPI (severity)	0.905 (0.574, 1.235)	< 0.001	0.544 (0.178, 0.911)	< 0.01
BPI (interference)	0.960 (0.667, 1.254)	< 0.001	0.764 (0.443, 1.084)	< 0.001

^^^ Abbreviations: CI, confidence interval; BMI: Body Mass Index; BPI: Brief Pain Inventory; MSK: Musculoskeletal;

^†^ Adjusted for age, gender, BMI and no. of comorbid diseases.

In the moderation analysis, we found that age, gender, and the number of comorbid diseases did not moderate the longitudinal relationship between chronic MSK pain and sleep. All of their interaction terms, namely, age × MSK pain (β = -0.001; 95% CI -0.170–0.168; P = 0.991); gender × MSK pain (β = 1.676; 95% CI -0.547–3.898; P = 0.139); and the number of comorbid diseases × MSK pain (β = -0.164; 95% CI -0.901–0.573; P = 0.662) did not have any statistical significance (Table 5).

**Table 5:** The relationship between MSK pain and insomnia through the moderators (n = 498) ^.

	Model	β	SE	P-value	95% CI
a. The effect of MSK pain on insomnia through age^†^	Constant	0.455	5.658	0.936	− 10.662, 11.572
	MSK pain (X)	1.791	6.009	0.766	− 10.014, 13.597
	Age (X xW1)	0.015	0.076	0.845	− 0.135, 0.165
	Interaction (X × W1)	− 0.001	0.086	0.991	− 0.170, 0.168
b. The effect of MSK pain on insomnia through gender)^‡^	Constant	1.186	3.084	0.701	− 4.874, 7.247
	MSK pain (X)	0.689	0.901	0.444	− 1.080, 2.459
	Gender (W2)	0.227	0.956	0.813	− 1.652, 2.105
	Interaction (X × W2)	1.676	1.131	0.139	− 0.547, 3.898
c. The effect of MSK pain on insomnia through no. of comorbid diseases^§^	Constant	0.056	3.225	0.986	− 6.281, 6.394
	MSK pain (X)	2.310	1.452	0.112	− 0.543, 5.163
	No. of comorbid diseases (W3)	0.112	0.352	0.751	− 0.580, 0.804
	Interaction (X × W3)	− 0.164	0.375	0.662	− 0.901, 0.573

^Abbreviations: β, beta coefficient; SE, standard error; CI, confidence interval; BMI: Body Mass Index; MSK: Musculoskeletal;

^†^Adjusted for gender, BMI, no. of comorbid diseases and insomnia at baseline;

^‡^Adjusted for age, BMI, no. of comorbid diseases and insomnia at baseline;

^§^Adjusted for age, gender, BMI and insomnia at baseline.

A sensitivity analysis was conducted to analyze the demographics and baseline scores between those followed up and missed. We found that participants of older age (OR 0.962, 95% CI 0.939- 0.986, P < 0.01) and higher BMI (OR 0.950, 95% CI 0.908–0.994. P < 0.05) were more likely to be missed. (Table 6).

**Table 6:** Sensitivity analysis of patients with or without follow up measures^ (Mean ± standard deviation (SD)/count (col %)).

	Patients with follow up measures (n = 498)	Patients without follow up measures (n = 220)	OR (95% CI)^†,^^Ψ^	P-value
Age (years)	69.19 ± 6.26	71 ± 7.35	0.962 (0.939, 0.986)	< 0.01
Gender (male = ref.)	134 (26.9%)	67 (30.5%)	1.162 (0.804, 1.679)	0.424
BMI (kg/m^2^)^‡^	23.96 ± 3.55	24.59 ± 3.47	0.950 (0.908, 0.994)	< 0.05
No. of comorbid diseases	4.18 ± 1.85	4.25 ± 1.85	1.011 (0.922, 1.110)	0.811
Insomnia (score)	11.62 ± 4.85	11.30 ± 5.36	1.008 (0.974, 1.043)	0.668
MSK pain (No = ref.)	107 (21.5%)	42 (19.1%)	0.821 (0.538, 1.251)	0.359

^^^Abbreviations: BMI: Body Mass Index; MSK: Musculoskeletal; OR: Odds ratio; CI: Confidence interval;

^†^Patients with or without follow up measures was categorized 1 or 0 (reference);

^Ψ^Multiple logistic regression;

^‡^There is a missing data because one participant is a wheelchair user and his body weight cannot be measured.

## Discussion

We confirmed that chronic MSK pain is very common among older people. Our baseline prevalence rate of 78.5% is comparable to previously reported rates of 60% to 75% among people over the age of 65 years^21,22^. Similar to the findings of other cross-sectional studies^11^, participants with chronic MSK pain have a higher degree of sleep impairment at baseline than those without pain, as indicated by the ISI scores, which met the definition of moderate clinical insomnia^23^.

Participants in this cohort all demonstrated improvement of the ISI at 18 months. This could not be fully explained by sampling error or regression to mean phenomenon. Instead, it was because participants in the cohort received the elderly health services under the charity program, which included a variety of health education seminars, exercise classes and cognitive trainings. However, despite receiving these kinds of complex interventions, our findings still support the hypothesis that the presence of chronic MSK pain is associated with the severity of insomnia in a longitudinal direction. Comparing to the “no pain-no pain” group, insomnia was worse in the “pain-pain” group, over the 18-month period. We did not detect any statistical significance between the “no pain-pain” and “no pain-no pain” groups; this may be due to the onset and duration of pain not being recorded, thus the impact on sleep impairment could not be fully analyzed. Nevertheless, the magnitude of improvement in the ISI over 18 months was smallest in the “pain-pain” group, followed by “no pain-pain”, “pain-no pain” and “no pain-no pain” groups, in ascending order. Similar to other studies, we confirmed that the female gender and baseline insomnia predict the severity of insomnia in a longitudinal direction^24^.

We also verified that greater pain severity and interference are associated with a higher degree of insomnia^25^. The pathophysiological process of how pain affects sleep has long been discussed; experimental studies have shown that the induction of nociceptive stimuli alters the microstructure of sleep but not the macrostructure of sleep^26,27^. Patients with chronic pain have been shown to experience alpha (α)-δ sleep anomaly, an EEG sleep pattern described α-like waves (thought to be a waking rhythm) superimposing upon the more normal δ waves during non-REM sleep^28,29^. That is, normal restorative deep sleep was interrupted by periods of mini-arousal or temporary wakening^30^. The elevated level of inflammatory cytokines in chronic pain conditions, such as interleukin (IL)-1β, IL-6, IL-10, and tumor necrosis factor-α, has shown to be associated with sleep disturbance^31,32^. Pain pathologies and the associated affective disorders also contribute to the severity of sleep impairment^33^. Furthermore, functional impairment as a result of pain also limits the degree of physical activity^3^, and low physical activity levels have shown to predict the incidence and prevalence of late‐life insomnia^34^.

Although a large number of cross-sectional studies have suggested that age, female gender, and co-morbid illnesses are associated with sleep impairment^3,19,35^, our findings have shown that they did not moderate the relationship in a longitudinal direction. These findings are novel and important, suggesting that chronic MSK pain itself is a strong predictor for insomnia. Further studies with large sample size will help to confirm this observation^36^. Nevertheless, the suggestion of regular exercise and the cautious use of analgesic medications will be able to improve pain and subsequent insomnia^37^.

Our study has some limitations. First, there was 30.6% missing data; although participants who were older and more obese were more likely to be missed, these two variables were not shown to be associated with the severity of ISI. Second, insomnia was measured subjectively by the ISI; objective assessment by polysomnography was not used in this cohort. Third, we did not study the effects of individual co-morbid affective disorders on chronic pain and sleep. Fourth, we did not consider the medications used in this population, which may have important influence over sleep quality. Finally, our data came from a cohort of older people in the primary care setting; it is possible that the results cannot be generalized to otherwise healthy elderly, or to older people in nursing homes.

## Methods

This study was conducted within the framework of a community charity program, which provide complex health services to elderly participants with multimorbidity^38^. Ethics approval was obtained from The Joint Chinese University of Hong Kong – New Territories East Cluster Clinical Research Ethics Committee. Written informed consent was obtained from all participants. All methods were carried out in accordance with relevant guidelines and regulations.

### Study design

An 18-month prospective cohort study.

### Participants and settings

Participants were recruited through the 8 primary health care clinics in the New Territories East region of Hong Kong, between December 2016 and October 2017. Participants eligible for enrollment in our study were over ≥ 60 years of age, with two or more concurrent chronic conditions, and being able to communicate in Chinese (Cantonese or Mandarin)^38^.

### Independent variables

The predictor variable (X) was the presence or absence of chronic MSK at baseline, defined by pain lasting ≥ 3 months, including regional pain (joints, limbs, back, and/or neck), a degenerative joint condition such as osteoarthritis, and/or musculoskeletal complaints that fall under the “chronic primary pain” classification of the International Classification of Disease-11 (ICD-11). Other predictors include age, gender, body-mass index (BMI) and the number of comorbid diseases at baseline.

Patients who responded ‘Yes’ to having chronic pain were asked to complete the Brief Pain Inventory (BPI), a validated tool for assessing chronic, non-malignant pain^39^. The BPI assesses pain severity (over the past 24 h, classified as ‘current’, ‘worst’, ‘least’, and ‘average’), and interference, with the following 7 areas (mood, physical activity, work, social activity, relations with others, sleep, and enjoyment of life). Higher scores are indicative of increasing pain severity and interference with daily functions. The BPI includes a body figure to record the sites and locations of pain.

The moderator variables (W) include age, gender, and the number of comorbid diseases at baseline. The comorbid diseases included 43 conditions listed in the cohort, with hypertension, hyperlipidemia, diabetes mellitus and coronary artery disease being the commonest reported diseases^38^.

### Dependent variable

The outcome variable (Y) was the Chinese validated insomnia severity index (ISI) at 18 months, a 7-item questionnaire to measure the degree of insomnia. It assesses the level of disturbance to sleep pattern, consequences of insomnia, and the degree of concerns or distresses related to the sleep problem. It comprises of problems in initiating and maintaining sleep, early morning awakening, noticeability of the sleeping problems to others, interference with daily functioning, and level of distress and satisfaction with current sleep pattern^40^. The ISI provides good coverage for the diagnostic criteria for insomnia, as defined by the DSM‐IV and ICSD^23^. The presence or absence of pain and BPI scores were assessed at 18 months again.

### Statistical analysis

Descriptive data were reported as percentage or mean ± standard deviation. The chrematistic differences between the two groups at baseline were analyzed using independent t-test for continuous variables or χ^2^ test for categorical variables, respectively. Linear regression models were used to identify significant variables associated with the ISI (continuous variable) at 18 months. The transition of MSK pain from baseline to 18 months was categorized into 4 groups (no pain–no pain; no pain–pain; pain–no pain; and pain–pain) and was transformed into dummy variables (no pain-no pain as reference group). The multivariate linear regression models were used to examine the relationships between the 4 groups, and the ISI changes over the 18 months. Subgroup analysis was conducted for the MSK pain group to evaluate the associations between the number pain sites, BPI-severity and BPI-interference scores. Moderation analysis was used to test any moderators including age (W1), gender (W2), and the number of comorbid diseases (W3); and moderate the relationship between pain (X) and ISI (Y) at 18 months. A significant moderation was determined as a statistically significant interaction term in each equation^41^. All models were adjusted for age, gender, BMI, the number of comorbid diseases, and ISI at baseline. Beta (β) coefficient and 95% CI were reported. Statistical significance of the variables was defined as P-value < 0.05. The analyses were performed using SPSS version 24 (IBM Corp, Armonk, NY).Sensitivity analysis was used to analyze missing data.

## Conclusions

In this longitudinal prospective study, chronic MSK pain predicts the severity of insomnia in older adults, and is not moderated by age, gender, and the number of comorbid diseases. Furthermore, pain severity and functional impairment are associated with a higher degree of insomnia. As such, our findings add evidence that the relationship between chronic pain and sleep is bidirectional, and it may be worthwhile to consider sleep as one of the core outcome measures in future pain trials. More research is needed to explain the overall mechanisms between chronic pain and sleep, which may potentially provide unique targets for intervention for both pain and sleep management. We also propose that pain management should be prioritized in the prevention of insomnia in older people.

## Supplementary Information

Supplementary Information.
